# INSPIRE: A phase III study of the BLP25 liposome vaccine (L-BLP25) in Asian patients with unresectable stage III non-small cell lung cancer

**DOI:** 10.1186/1471-2407-11-430

**Published:** 2011-10-07

**Authors:** Yi-Long Wu, Keunchil Park, Ross A Soo, Yan Sun, Karin Tyroller, David Wages, Guy Ely, James Chih-Hsin Yang, Tony Mok

**Affiliations:** 1 Guangdong General Hospital & Guangdong Academy of Medical Sciences, Guangzhou, China; 2 Samsung Medical Center, 50, Irwon-Dong, Gangnam-gu, Seoul, 135-710 Korea; 3 National University Cancer Institute Singapore (NCIS), National University Hospital, Singapore; Cancer Science Institute of Singapore, National University of Singapore, Singapore; 4 Cancer Hospital and Institute Chinese Academy of Medical Sciences, China; 5 Merck KGaA, Darmstadt, Germany; 6 EMD Serono, Rockland, USA; 7 National Taiwan University Hospital Department of Oncology, 4404 Room, 4F, No.1 Chang De St, 100 Taipei, Taiwan; 8 State Key Laboratory of Southern China, The Chinese University of Hong Kong, Sir YK Pau Cancer Center, Prince of Wales Hospital, Hong Kong, China

## Abstract

**Background:**

Previous research suggests the therapeutic cancer vaccine L-BLP25 potentially provides a survival benefit in patients with locally advanced unresectable stage III non-small cell lung carcinoma (NSCLC). These promising findings prompted the phase III study, INSPIRE, in patients of East-Asian ethnicity. East-Asian ethnicity is an independent favourable prognostic factor for survival in NSCLC. The favourable prognosis is most likely due to a higher incidence of EGFR mutations among this patient population.

**Methods/design:**

The primary objective of the INSPIRE study is to assess the treatment effect of L-BLP25 plus best supportive care (BSC), as compared to placebo plus BSC, on overall survival time in East-Asian patients with unresectable stage III NSCLC and either documented stable disease or an objective response according to the Response Evaluation Criteria in Solid Tumors (RECIST) criteria following primary chemoradiotherapy. Those in the L-BLP25 arm will receive a single intravenous infusion of cyclophosphamide (300 mg/m^2^) 3 days before the first L-BLP25 vaccination, with a corresponding intravenous infusion of saline to be given in the control arm. A primary treatment phase of 8 subcutaneous vaccinations of L-BLP25 930 μg or placebo at weekly intervals will be followed by a maintenance treatment phase of 6-weekly vaccinations continued until disease progression or discontinuation from the study.

**Discussion:**

The ongoing INSPIRE study is the first large study of a therapeutic cancer vaccine specifically in an East-Asian population. It evaluates the potential of maintenance therapy with L-BLP25 to prolong survival in East-Asian patients with stage III NSCLC where there are limited treatment options currently available.

**Study number:**

EMR 63325-012

**Trial Registration:**

Clinicaltrials.gov reference: NCT01015443

## Background

The incidence of lung cancer is high in Asia, particularly Eastern Asia, and is on the rise. There were an estimated 873,300 new cases in Asia in 2008 and 753,800 deaths from lung cancer that same year [1,2]. The rising incidence is thought to reflect smoking behaviours among Asian men while other factors are thought to be largely responsible for the rise among women, including cooking oil vapour, coal burning and outdoor air pollution [3]. Non-small cell lung cancer (NSCLC) accounts for approximately 80-85% of all cases of lung cancer [4] and a substantial proportion of patients with NSCLC are initially diagnosed with stage III disease [5].

Concurrent chemotherapy and radiotherapy is generally considered the standard of care for unresectable stage III NSCLC [6-9]. Local and distant treatment failures are common among patients with stage III NSCLC, and the majority die within three years of diagnosis. Chemoradiotherapy may also be associated with substantial toxicity, including myelosuppression, oesophagitis, nausea and vomiting. Therapeutic progress using chemoradiotherapy appears to have reached a plateau and therefore new treatments are urgently needed [10,11].

Mucin 1 (MUC1) is a glycoprotein that was first identified as a tumour-associated antigen in the mid-1980s; it is overexpressed and aberrantly glycosylated in many carcinomas including NSCLC. MUC1 can stimulate cell proliferation and suppress apoptosis and therefore may have a role in tumour progression. Moreover, abnormal MUC1 expression is associated with progressive disease and metastasis [12,13].

BLP25 liposome vaccine or L-BLP25 (Stimuvax^®^) is a therapeutic cancer vaccine that targets the MUC1 antigen. A phase II study comparing L-BLP25 plus best supportive care (BSC) with BSC alone in 171 patients with stage IIIB/IV NSCLC reported median overall survival times of 17.4 months and 13 months, respectively, after a median follow-up of 26 months (adjusted hazard ratio [HR] 0.739, 95% confidence interval [CI] 0.509-1.073, *p *= 0.112). The greatest difference was observed in patients with stage IIIB locoregional disease (n = 65) in whom the median survival time had not been reached for the L-BLP-25 arm, compared with 13.3 months for the BSC arm (adjusted HR = 0.524, 95% CI 0.261-1.052, *p *= 0.069) at the time of the initial publication [14]. An updated survival analysis in the patients with stage IIIB locoregional disease reported a median survival time of 30.6 months with L-BLP25 vs 13.3 months in the control arm (follow up of 53 and 57 months, respectively; HR 0.548, 95% CI 0.301-0.999) [15].

On the basis of these findings, the phase III trial, START (**S**timulating **T**argeted **A**ntigenic **R**esponses **T**o NSCLC), was initiated. START is investigating the efficacy and safety of L-BLP25 as maintenance therapy for unresectable stage III NSCLC in a global population of patients from North and South America, Europe and Australia. Recruitment for the START study is expected to be completed in 2011.

Asian ethnicity has been consistently shown to be an independent favourable prognostic factor for survival in patients with NSCLC. Data from the FLEX study investigating cetuximab plus chemotherapy versus chemotherapy alone, and the INTEREST study of gefitinib versus docetaxel, indicated that Asian patients with advanced NSCLC had longer survival compared with white patients (FLEX) and non-Asian patients (INTEREST) [16,17]. Furthermore, in a meta-analysis of randomised controlled trials of first-line chemotherapy in advanced stage NSCLC, a longer overall survival was seen in Asian compared with Caucasian patients [18]. The association between Asian ethnicity and survival is independent of smoking status and other prognostic factors [19-21]. The likely underlying explanation is a high incidence of polymorphism in the epidermal growth factor receptor (EGFR) gene among East-Asian populations, with a prevalence of EGFR mutations of 25-35% compared with around 10% in other ethnic groups [22-24]. A high level of EGFR protein may be a poor prognostic factor for survival in NSCLC [25].

Because the longer median survival of Asian patients with NSCLC could be a confounding effect in a global trial, INSPIRE (Stimuvax trial **I**n Asian **NS**CLC **P**atients: stimulating **I**mmune **Re**sponse), was initiated in a solely East-Asian population to complement the START trial. In addition, a smaller study is ongoing in Japan. The INSPIRE study will evaluate the efficacy and safety of L-BLP25 as maintenance therapy for East-Asian patients (excluding Japan) with unresectable stage III NSCLC. Together, INSPIRE and START will enrol approximately 2,000 patients. Both studies have survival as the primary endpoint; progression is assessed by the investigator according to institutional standards.

## Methods/Design

### Patients

All patients in the INSPIRE study have locally advanced unresectable stage III NSCLC [26], and documented stable disease or an objective response according to the Response Evaluation Criteria In Solid Tumors (RECIST) criteria following primary chemoradiotherapy (completed from 4-12 weeks before randomization). Patients with metastatic disease or malignant pleural effusion (MPE) are excluded from the study. The inclusion and exclusion criteria are summarized in Table 1. Recruitment for the INSPIRE study is currently underway in approximately 40 trial sites in China, Hong Kong, Singapore, South Korea, and Taiwan with a target study population of 420 patients. Written informed consent must be provided by all patients before any trial-related activities are carried out.

**Table 1:** Key inclusion and exclusion criteria

**Inclusion criteria**	Histologically documented unresectable stage III NSCLC, with stage confirmed by imaging (CT and/or MRI and/or PET)
	Completion of chemoradiotherapy (concomitant or sequential) ≥ 4 weeks and ≤ 12 weeks prior to randomization, consisting of ≥ 2 cycles of platinum-based chemotherapy and a radiation dose of ≥ 50 Gy
	Stable disease or objective response after primary chemoradiotherapy according to RECIST [37] documented ≤ 4 weeks prior to randomization
	ECOG performance status of 0 or 1
	Platelet count ≥ 140 × 10^9^/L; WBC ≥ 2.5 × 10^9^/L; haemoglobin ≥ 90 g/L
	≥ 18 years of age
**Exclusion criteria**	
*Prior therapies*	Lung-cancer-specific therapy (including surgery) other than primary chemoradiotherapy
	Immunotherapy ≤ 4 weeks prior to randomization
	Investigational systemic drugs ≤ 4 weeks prior to randomization
*Disease status*	Metastatic disease
	Malignant pleural effusion
	Past or current history of neoplasm other than lung carcinoma*
	Autoimmune disease or recognized immunodeficiency
	Pre-existing medical conditions requiring chronic steroid or immunosuppressive drug therapy
	Signs and symptoms suggestive of, or family history of, transmissible spongiform encephalopathy
	Clinically significant active or chronic infectious hepatitis
	Hepatic dysfunction (ALT > 2.5 × ULN, AST > 2.5 × ULN or bilirubin ≥ 1.5 × ULN)
	Renal dysfunction (serum creatinine ≥ 1.5 × ULN)
	Clinically significant cardiac disease
	Splenectomy

*Except for curatively treated nonmelanoma skin cancer, in situ carcinoma of the cervix, or other cancer curatively treated and with no evidence of disease for at least 5 years

ALT: alanine aminotransferase; AST: aspartate aminotransferase; CT: computed tomography; ECOG: Eastern Cooperative Oncology Group; MRI: magnetic resonance imaging; NSCLC: non-small cell lung cancer; RECIST: Response Evaluation Criteria In Solid Tumors; ULN: upper limit of normal; WBC: white blood cell count.

### Study design and treatments

**Figure 1:**
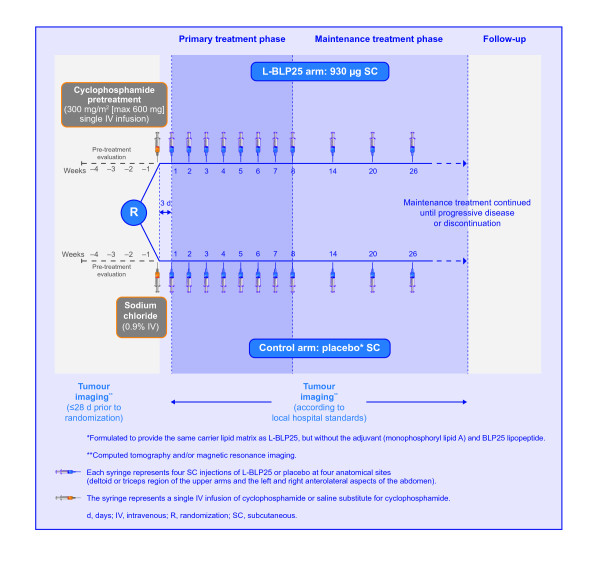
**Study design**.

The design of the INSPIRE study is summarized in Figure 1. INSPIRE is a double-blind, placebo-controlled, phase III clinical trial in which patients are randomized in a 2:1 ratio to either L-BLP25 or placebo. Randomization to treatment is stratified by disease stage at first diagnosis (IIIA versus IIIB), histology (adenocarcinoma versus non-adenocarcinoma; Asian patients with adenocarcinoma have been shown to have a better prognosis than patients with other types of histology [27]) and type of primary chemoradiotherapy (concomitant versus sequential). All patients will receive BSC, which may include, but is not limited to, psychosocial support, nutritional support and other supportive therapies.

Three days before the first L-BLP25 vaccination, a single intravenous infusion of cyclophosphamide 300 mg/m^2 ^(maximum dose 600 mg) (L-BLP25 arm) or saline (control arm) will be administered. The purpose of administering cyclophosphamide is to inhibit T_reg _cells and enhance the development of an effective response to the immunogen of the vaccine [28]. The primary treatment phase consists of 8 subcutaneous vaccinations of L-BLP25 930 μg or placebo at weekly intervals. L-BLP25 is composed of a lyophilized preparation of the 25-amino-acid BLP25 lipopeptide, an immunoadjuvant (monophosphoryl lipid A) and three lipids (cholesterol, dimyristoyl phosphatidylglycerol and dipalmitoyl phosphatidylcholine). The nominal dose of L-BLP25 is different to that previously stated [14,29]. In clinical studies prior to 2008 the density determination of L-BLP25 prior to freeze-drying of the final powdered product led to the content of L-BLP25 being declared as 1000 μg. However, the more recent density determination means that the majority of phase IIb study participants actually received 930 μg antigen as opposed to the 1000 μg that was declared, and phase III studies including INSPIRE continue to use the 930 μg dose. The placebo, which is also delivered subcutaneously, is formulated to provide the same carrier lipid matrix as the L-BLP25 vaccine, but without the monophosphoryl lipid A adjuvant and BLP25 lipopeptide. L-BLP25 can be associated with local injection site reactions; however, as these reactions tend to be mild, and are not universal, patients and physicians cannot definitively determine who receives placebo or vaccine.

The primary treatment phase is followed by a maintenance treatment phase, with vaccinations at 6-week intervals until disease progression or discontinuation from the study. As the investigators will evaluate disease progression according to institutional standards, the technique and timing of imaging will vary from site to site. Although patients with disease progression will no longer receive either the study medication or placebo, where possible they will remain in the trial for the evaluation of the primary endpoint, overall survival time. With the exception of the trial treatment and any necessary supportive treatment, patients enrolled in the study are not permitted drugs that are intended to modulate the immune system or systemic immunosuppressants. While receiving trial treatment, patients are not permitted any other investigational drugs, chemotherapy or radiotherapy.

Patients are followed up at each treatment visit during the primary (i.e. weekly) and maintenance (i.e. every 6 weeks) treatment phases, and 6 and 12 weeks after the last study treatment. Long-term follow-up will comprise assessments at 12-week intervals beginning 24 weeks after the last study treatment and continuing until death or study end.

### Study objectives

The objectives of the study are presented in Table 2. The primary objective of the INSPIRE study is to assess the treatment effect of L-BLP25 plus BSC, as compared to BSC alone, on overall survival time. Secondary objectives include the evaluation of differences between treatment arms in terms of time to symptom progression (based on the Lung Cancer Symptom Scale [LCSS] questionnaire), time to progression, progression-free survival time and safety parameters. Adverse events of special interest in the trial include injection-site reactions, thrombocytopenia, hepatic dysfunction and autoimmune disease.

**Table 2:** Study objectives

**Primary objective**	To assess the treatment effect of L-BLP25 plus BSC, as compared to BSC alone, on overall survival time
**Secondary objectives**	Time to symptom progression (based on LCSS questionnaire)
	Time to progression
	Progression-free survival time
	Time to treatment failure
	Safety
**Other objectives**	Quality-of-life (EQ-5D/LCSS)
	Healthcare resource utilization and work status
	Biomarkers/pharmacogenetics

BSC: best supportive care; EQ-5D: EuroQol Group 5-Dimension questionnaire; LCSS: Lung Cancer Symptom Scale.

### Statistics

The sample size of 420 patients was chosen to allow for the potential identification of a trend towards prolonged survival with L-BLP25 while taking the following factors into account: a 2:1 randomization ratio (L-BLP25 vs control arm), an increase of median overall survival time from 24 months in the control arm to 31 months with L-BLP25, a linearly increasing cumulative enrolment over 30 months and a total trial duration of 54 months (follow-up of 24 months). Follow-up will be event-driven with analysis to take place after 211 deaths are reported.

### Ethical considerations

Prior to initiation of the study, each of the participating sites must obtain local ethics committee approval from the appropriate body. All research will conform to the Declaration of Helsinki, as well as local legal and ethical requirements.

## Discussion

Previous research reported longer survival times with L-BLP25 plus BSC versus BSC alone in patients with stage IIIB/IV NSCLC, and this effect was more pronounced in the subset of patients with stage IIIB locoregional disease [14,15]. These findings prompted the initiation of the START and INSPIRE studies to evaluate a potential survival benefit with L-BLP25 in a larger population with stage III NSCLC.

The most common adverse events with L-BLP25 treatment are Grade 1 flu-like symptoms (such as fatigue), minor injection site reactions (such as erythema and bruising) and adverse events related to cyclophosphamide administration (most commonly nausea). The pattern and incidence of adverse events does not appear to change over time with long-term treatment [14,29-32].

In ongoing trials, events that could be suggestive of autoimmune disease were uncommonly observed. In March 2010 a case of encephalitis was assessed as a suspected unexpected serious reaction. As a result, the clinical program for L-BLP25 in all recruiting studies worldwide was temporarily suspended. This decision was taken in alignment with the FDA's clinical hold placed on the Investigational New Drug application for L-BLP25. This case was observed in a mechanism of action study of L-BLP25 in multiple myeloma. A potential autoimmune causality of this case could not be excluded. Subsequent work-up and overall safety analysis of L-BLP25 in NSCLC led to the clinical hold being lifted and trials of L-BLP25 in NSCLC have restarted with additional safety monitoring procedures in place, such as more frequent neurological examinations of patients during the study and closer monitoring of patients should they develop neurologic signs of any origin.

There is an urgent need for new treatments in patients with unresectable stage III NSCLC. With chemoradiotherapy, 5-year survival rates for these patients are less than 15% [33]. The populations of both the INSPIRE and START studies are limited to patients with unresectable stage III disease. There is a possibility that the radiotherapy which was received by the majority of patients with stage IIIB locoregional NSCLC may have contributed to the benefit seen in the phase II trial of L-BLP25. Radiotherapy induces pro-immunogenic effects that may render tumours more responsive to subsequent immunotherapy [34,35]. All patients in the INSPIRE study will have been treated with radiotherapy as well as chemotherapy before being randomized into the study. The control arm of the trial is reflective of the global standard of care received by patients with stage III disease. The placebo used in the control arm has neither the adjuvant nor the BLP25 antigen. The adjuvant was excluded from the placebo, given that even if the adjuvant was capable of eliciting a non-specific immune response in the absence of the BLP25 antigen, it is highly unlikely that any non-specific response would lead to an improvement in survival. As the placebo does not contain the peptide either, the placebo is very unlikely to elicit an immune response or influence overall survival time.

The INSPIRE study uses the 6^th ^edition of the staging system published in 2002 by the Union Internationale Contre le Cancer (UICC) and the American Joint Committee on Cancer (AJCC) [26]. In the 7^th ^edition, published in 2009, malignant pleural effusion now constitutes metastatic disease and as such is stage IV [36]. However, this will not impact on the INSPIRE study as malignant pleural effusion is an exclusion criterion.

The INSPIRE study, together with findings from the smaller phase I/II study of L-BLP25 also currently ongoing in Japan, will provide a valuable insight into the potential role of L-BLP25 as maintenance therapy for East-Asian patients with unresectable stage III NSCLC. These trials, alongside the START trial, will expand substantially upon the body of knowledge for the novel therapeutic cancer vaccine L-BLP25 and its clinical role in the treatment of unresectable stage III NSCLC. The three trials will also provide preliminary data on ethnic factors underlying the immune response to NSCLC following receipt of a therapeutic cancer vaccine.

## Declaration of competing interests

Merck KGaA funded the development of the article, including the article-processing charge. DW has received a salary from EMD Serono. GE has received funding from Merck Serono. TM has received funding from AstraZeneca, Roche, Merck Serono and Eli Lilly. The other authors declare that they have no competing interests.

## Authors' contributions

All authors have been involved in critically revising the drafts of the manuscript and read and approved the final manuscript. TM was involved in manuscript drafting. All authors have been involved in the development of the study design.

## Pre-publication history

The pre-publication history for this paper can be accessed here:

http://www.biomedcentral.com/1471-2407/11/430/prepub
